# Effect of Integrated Physical Activities with Mathematics on Objectively Assessed Physical Activity

**DOI:** 10.3390/children5100140

**Published:** 2018-10-11

**Authors:** Spyridoula Vazou, Pedro F. Saint-Maurice, Miriam Skrade, Gregory Welk

**Affiliations:** Department of Kinesiology, Iowa State University, Ames, IA 50011, USA; pedro.saintmaurice@nih.gov (P.F.S.-M.); mbskrade@gmail.com (M.S.); gwelk@iastate.edu (G.W.)

**Keywords:** classroom, movement integration, exercise, accelerometer, moderate-to-vigorous physical activity

## Abstract

Background: One of the promising strategies for increasing physical activity (PA) at school is to integrate it with academic learning. The purposes of this study were: (a) to examine differences in objectively measured PA levels between integrated PA with mathematics and traditional lessons, and (b) to evaluate the PA levels of different integrated PAs. Methods: Seventy-seven 4th grade students (41 males) were included in an intervention (Move for Thought program: M4T) group (*n* = 46) that utilized PA integrated with mathematics or a control group (*n* = 31). Accelerometer data from each student were collected during five complete school days. M4T and control classroom sessions were identified using teachers’ logs. Accelerometer data were extracted, processed separately, and aggregated into a single data set. Minutes and percent time at different PA intensities were obtained using accelerometer minute-by-minute predicted METs. Results: One-way ANOVAs on PA levels showed a significant group effect (*F* = 5.33, *p* < 0.05) on moderate-to-vigorous PA (MVPA) in favor of the M4T group, but not on sedentary and light PA. The most active integrated PA provided 10.88 min of MVPA (*SD* = 11.87; 21.38 ± 24.38%) in a 50 min class period. Conclusion: Integrating PA with mathematics in the classroom can contribute to increasing MVPA levels in children.

## 1. Introduction

For children to meet the recommended guidelines for daily physical activity (PA), national organizations promote the adoption of a whole-of-school approach by accumulating PA throughout the school day, across different settings (e.g., physical education, recess, classroom, before- and after-school) [1,2]. One setting that has received increasing interest from both researchers and educators is the academic classroom [3,4,5,6]. Physical activity in the classroom is usually implemented as a short activity break (mainly as an energy booster) or through integration with academic subjects (to teach or practice academic concepts); it varies in intensity, and it typically lasts between 5 and 15 min [3,5]. Integrating PA with academic learning may be a more feasible strategy to help children meet the recommended PA guidelines, compared to extended recess or other strategies, because educators do not feel that they are taking time out of academic content during the already busy school day [6,7].

An evolving literature shows that PA in the classroom has the potential to contribute to the PA levels of children, in addition to the benefits on cognitive and academic outcomes [8,9,10,11], however, results remain inconclusive due to limited high quality interventions [12]. A number of intervention studies conducted in the academic classroom have also only assessed overall PA at school [13,14,15,16] or overall PA levels during the week [17,18,19]. To more objectively evaluate this approach it is important to directly capture activity levels at the classroom level. Another gap in the literature is the lack of studies evaluating the PA levels of lessons that utilized integrated learning activities and the qualitative characteristics of those lessons. Considering the large number of classroom-based intervention studies that have been identified from recent reviews and meta-analyses [4,5,6], only 9 studies have objectively estimated the PA of lessons integrated with learning [13,14,15,16,18,20,21,22,23]. Of these, five compared the PA levels of the integrated lessons to a control group or condition [14,18,20,21,22], and only three provided detail on the actual intensity of the integrated activities [18,20,22]. The “Virtual Traveler” program [18] embedded Google earth videos on interactive whiteboards and children reached MVPA levels by simulating appropriate on-the-spot movements as they ‘traveled’ to, and interacted with, locations. Grieco and colleagues [20] used one 15-minute integrated PA lesson (named “spelling relay”) in their study. In the low to moderate intensity group, elementary school students walked for the relay whereas, in the MVPA group, students combined running and jumping. The third study was conducted with preschoolers [22], using a combination of observations and accelerometer data. Results showed that preschoolers in the integrated PA classroom were significantly more likely than preschoolers in the control classroom to exhibit MVPA during indoor circle time and free-choice time (both indoors and outdoors), whereas transitions and snack time did not differ between groups [22]. These are the only studies to our knowledge that have objectively assessed the PA levels of different types of integrated PAs during class time. Therefore, it is evident that research on the activity level of integrated PAs in the classroom remains scarce.

The purpose of this study was to examine whether children who were engaged in PAs integrated with mathematics, during one lesson, would be more physically active or not, compared to children who attended a traditional academic lesson of the same duration. A second purpose was to obtain an objective assessment of the PA levels of various integrated PAs from the Move for Thought (M4T) program. The effectiveness of the M4T program on math performance over an 8-week intervention has been published elsewhere [24].

## 2. Materials and Methods

### 2.1. Participants

Seventy-seven 4th grade students (*M* age = 9.42 ± 0.50; 41 males and 36 females; body mass index = 17.74 kg/m^2^ ± 2.80) from one elementary school in a rural area of a Midwestern state (United States) were included either in an intervention (*n* = 46) or control (*n* = 31) group. The study was approved by the Institutional Review Board at Iowa State University (IRB id: 12-499). Parental consent and student assent were collected before data collection. The present sample of participants represents a convenience subsample of a larger intervention study that focused on the role of integrated PA with mathematics on math performance and motivation [24]. The sample in this study was the only group that wore monitors during classroom time throughout the M4T intervention.

### 2.2. Instruments

The SenseWear Armband (SWA) (BodyMedia, Inc., Pittsburgh, PA, USA) is a wireless pattern-recognition device that integrates motion sensor data with a variety of heat-related sensors, and demographic variables to estimate energy expenditure (EE) and minutes of PA [25]. This monitor has been tested in children and has been shown to provide accurate estimates of PA and EE in this population [26,27,28]. The multisensor nature of the monitor provides advantages over traditional accelerometry-based monitors and can contribute to more accurate estimates of lower intensity activities [29]. An additional advantage of the SWA for field-based research is that it automatically detects nonwear time. The SWA was initialized with 1-min epochs and data were downloaded using InnerView v6.1 software (BodyMedia, Inc., Pittsburgh, PA, USA).

### 2.3. Design and Procedure

After the M4T project started, a random intervention week was selected, after communication with teachers, in which participants wore the SWA monitors. Participants were provided with instructions on how to wear the SWA monitor before data collection. The SWA monitors were worn throughout the school day, placed in the morning and collected by the teachers at the end of each school day. The monitors were collected seven days later by the research team. The mathematics lessons that were integrated with PA based on the M4T kit were identified based on teacher’s logs obtained from the broader study [24] and data were analyzed for those specific lessons. During the same week, the identical time periods were identified for the control classes (traditional lessons without integration of PA with academic learning). The duration of both lessons was 50 min.

### 2.4. Move 4 Thought (M4T) Activities

The M4T activities were developed to be integrated with academic subjects in the classroom and typically lasted approximately ten minutes. The M4T kit, which is freely available on the website of Iowa Department of Education, includes ten activities that can be adapted for any elementary grade and for any subject area. The activities were designed to allow instruction to remain focused on the academic lesson while students are safely physically active within the limited space of the classroom. The teachers were encouraged to use one M4T activity per day in integration with mathematics for about 10 min and had the autonomy of selecting when and how to use the activities (e.g., the ones that were most preferable to the students or themselves or fit best with the content of the lesson they were teaching). The 10 min duration was recommended because it seems both feasible and effective, according to the literature [3]. Eight out of the ten M4T activities were used in the classroom during the week that the PA data were collected. Two of those activities were performed individually; the first one by having the students stand on their self-space and jump the answer to a problem the teacher provides (“Jump the Answer”) and the second by moving around the classroom, picking a card with a problem and providing the answer as the teacher passes by (“Move Around”). For the remaining six activities, the students were working as a group (“Curious Ball”, “To the Wall”, “Over/Under”, “Messed-up Train”) or with a partner (“Red Light”, “Find your Pair”) to meet group activity challenges. The description and characteristics of the implemented M4T activities are presented in Table 1.

### 2.5. Data Processing and Analysis

Accelerometer data from each student were collected during five complete school days. Students were being supervised (the monitors were placed and collected by the teachers and at the end of each school day), therefore, nonwear compliance criteria were not used. Accelerometer data during the M4T and the matched control lessons were extracted, processed separately, and aggregated into a single data set. The activity time was calculated as a percentage of the whole class time, which was 50 min. Students’ minutes and percent time at different PA intensities were obtained using accelerometer minute-by-minute predicted METs and categorized as follows: sedentary (if METs <2.0), light (if METs >2.0 and <4.0), moderate (if METs >4.0 and <6.0), vigorous (if METs >6.0), and moderate to vigorous (MVPA) (if ≥4.0 METs). The names of the M4T activities that were implemented in the intervention lesson (one activity per lesson) were identified from preexisting teacher logs and were coded for all accelerometer data. For the primary purpose of the study, two one-way analysis of variance (ANOVA) on minutes and percentage of PA levels (sedentary, low, moderate, vigorous PA, MVPA), with two groups (intervention, control) for the between-subject factor, were conducted. Second, to examine the PA levels of different academic integrated PAs from the M4T program, descriptive statistics were used. Data were processed using SAS v9.2 (SAS Institute, Cary, NC, USA) and were analyzed using the Statistical Package for Social Sciences (IBM SPSS Statistics 23).

## 3. Results

### 3.1. Comparison of PA Levels Between Groups

One-way ANOVAs on PA levels showed a significant group effect on minutes of MVPA [*F*(1,75) = 5.87, *p* = 0.018] and percentage in MVPA [*F*(1,75) = 5.33, *p* = 0.024], but not on sedentary and light PA levels. Students in the M4T group spent significantly more time in MVPA during a class period (7.08%) compared to the control group (2.84%). This effect appeared primarily due to moderate intensity PA (5.95% for the intervention group, 2.31% for the control group, *F*(1,75) = 5.63, *p* = 0.020) rather than to vigorous intensity PA (1.12% for the intervention group, 0.53% for the control group, *F*(1,75) = 1.18, *p* = 0.281). Descriptive statistics and effect sizes between the two groups are presented in Table 2. Comparisons based on gender and BMI were also conducted with no significant differences between groups.

### 3.2. Assessment of PA Levels of M4T Activities

Descriptive statistics on minutes and percent time in sedentary, light, and MVPA for the M4T activities are presented in Table 3. The average wear time for the integrated lessons was 49.81 min (*SD* = 1.97). There is notable variability in the minutes of MVPA among activities with the most active math-integrated M4T activities, providing 10.88 min of MVPA (“Find your Pair”), and the least active, providing only approximately one minute of MVPA (0.84 min for “Jump the Answer” and 1.07 min for “Move Around”). However, the two aforementioned activities had the longest duration of light PA (29.12 min and 31.27 min, respectively). The remaining five math-integrated M4T activities provided on average five minutes of MVPA (3.83 min to 7 min) per activity per academic lesson.

## 4. Discussion

The primary aim of this study was to objectively compare the PA levels of students exposed to integrated PA with math lessons compared to those exposed to traditional lessons. It was found that the math lessons with integrated M4T activities in the elementary classroom accumulated significantly more minutes of MVPA in the class compared to the traditional lessons (with a medium effect size). This finding is consistent with the results from previous interventions that have reported higher levels in MVPA during academic lessons compared to control [13,14,18,20,21,22]. It should be noted that even though the difference in MVPA was significant, the actual average duration that children were in MVPA during the math lesson with integrated PA was 3.45 min, compared to 1.26 min of MVPA that was accumulated in the traditional lesson. A similar difference has also been found in intervention studies that integrated PA with mathematics (3.6 min of MVPA in lesson time after 6 weeks of program implementation) [14] and with mathematics and English (2.3 min of MVPA after an active virtual field trip) [18]. However, even though the overall difference was small the effect size was medium; therefore, it should not be automatically considered as “negligible” or “unimportant” from a practical standpoint. Previous studies with a similar difference on MVPA also found a significant effect of the intervention on on-task behavior [14,18], which has been shown to be a significant predictor of academic success [30]. The 8-week M4T intervention also had a significant impact on mathematic performance [24]. Therefore, the potential benefit of integrated PA with academic learning extends beyond the small increases on PA levels as it works synergistically in promoting school performance. In our study the overall difference in MVPA during the PA data collection is not surprising for several reasons. The teachers were given the autonomy to select whether, when, and how to integrate the PAs in their mathematics lessons and choose the teaching practices that were most effective for their students, instead of being constrained to follow a regimented experimental protocol. In addition, the teachers were encouraged to use only a small portion of their lesson (10 min) to integrate PA with mathematics; this time also counted for instruction and for checking the answers of the students. Lastly, no incentives were provided to strengthen participation because we wanted to increase external and ecological validity and evaluate the real-world implementation of the integrated PAs.

This study also provides a unique contribution to the literature by comparing the intensity levels of different activities designed to integrate movement with mathematics in the classroom. The results revealed considerable variation in the duration and intensity of the different integrated PAs. The M4T activity that elicited the highest proportion of lesson time (21.91%; 10.88 min) in MVPA was the “Find your Pair” activity. In this activity, students are constantly moving in the classroom in a movement pattern assigned by the teacher (e.g., skipping) with the goal to find the classmate with the matching card (answer or problem, respectively) and continue moving in place as a pair until the teacher checks all paired cards. This activity involved collaboration among classmates and minimized waiting time, characteristics that possibly have contributed to the higher levels of MVPA in the classroom. Likewise, a “competitive” relay that was integrated with academic learning [20] elicited similar levels (12.57 min) of MVPA. However, it should be noted that competitive games might elicit positive or negative emotions and various levels of student engagement based on how students perceive their competence related to others. If the motivational climate is focused on who is exhibiting superior ability over others or prioritizes winning then it is possible that students may feel anxiety and avoid participation, whereas motivational climates that focus on collaborative effort and personal best (without overemphasizing winning) are more likely to elicit positive feelings and higher levels of engagement [31,32]. 

In this study, the majority of the integrated M4T activities provided, on average, five added minutes of MVPA (3.83 min to 7 min; 5 activities). These activities were all performed with a group of students and were focused on group challenges or relays. Even in studies where the duration of the classroom-based PA varies, it has been shown that students accumulate similar levels of MVPA (4–4.3 min) as observed in our study, despite the different length of the conditions (5, 10, 20 min) [33]. Furthermore, the five-minute duration of activity breaks appears to be both feasible as well as preferable by teachers and students [33]. However, it remains unclear whether this duration is also preferable for integrating PA with academic learning because of the extra time that is typically needed to explain and practice the PA with the academic content. This research question remains to be further explored in future studies. 

Two of the M4T integrated PAs (“Move Around” and “Jump the Answer”) only accumulated approximately one min of MVPA. Both of those activities were performed individually in the classroom. It is possible that students might have felt uncomfortable being highly active in the limited classroom space while being observed by peers and the teacher whereas group tasks might have been more fun and less intimidating to fourth graders. However, this hypothesis needs to be tested, as previous studies have shown mixed results. Desk-based PAs in integration with learning resulted in 14 min of MVPA in one study [13] but only 2.3 min of MVPA in another [18]. It should be noted that both of the M4T activities with the shortest duration of MVPA had the longest duration of light PA (29.12 min and 31.27 min; 63% and 59%; respectively), meaning that the students were not sedentary for the majority of the classroom time, unlike other studies that have shown high levels of sedentary behavior in integrated PA lessons [14]. Therefore, academic learning with integrated PA of light intensity could possibly have a meaningful contribution to breaking sedentary behavior in the classroom. 

This study has several limitations that need to be considered. We recognize the study’s small size as an important limitation. Further, the findings summarized here should be considered applicable only to students that integrated math with PAs contained in the M4T kit. They cannot be assumed to generalize to other grades, academic subjects, or different types of classroom-based PAs without further study. Another limitation is that the activities were assessed only over a five-day period. Longer durations of objectively measured integrated PAs are essential in order to assess variations that may be due to the complexity of the academic content, as well as assess the sustainability of this novel approach over time. For example, different duration and intensity of integrated PAs may be needed when teachers teach new content compared to when they review already acquired knowledge.

## 5. Conclusions

To conclude, this study showed that integrating PA with mathematics in the academic classroom, using the freely available and easy-to-use “Move for Thought” kit, resulted in increased MVPA, compared to a traditional lesson. This positive finding, along with results from other studies, suggests that programs that integrate PA with academic learning have strong potential to help students accumulate minutes in MVPA within the academic classroom. Another unique contribution to the literature is the objective assessment of the duration and intensity of a variety of PAs integrated with mathematics and the inclusion of a control group. Even though it is reasonable to expect variability of MVPA within an integrated PA based on modifications made by teachers (e.g., on the subject area and the teaching objective) and the level of student engagement, this study provides a brief taxonomy of a number of PAs integrated with mathematics that has been missing in the literature. This new knowledge on the qualitative (e.g., content of activities, social environment) and quantitative characteristics of integrated PAs could contribute to understanding the underlying mechanisms of the relationship between PA and cognitive or academic achievement [34,35], evaluating the dosage of novel intervention programs [12], and supporting teachers in making decisions to better prepare their lessons and set clear expectations on their students.

## Figures and Tables

**Table 1:** Description and characteristics of implemented M4T activities.

M4T Activity	Description	Ind.	With Others	Self- Space	General Space	Equip.
1. Curious Ball	Students in a circle formation pass the ball to each other and provide answers as asked. After passing the ball they run around, outside the circle, and return to their initial position. Group challenge: “how many passes can you do within 1 min?”.		x		x	Ball
2. Move Around	On a “go” signal, students move around the classroom. On a “Stop” signal, they pick up a card from a pile closest to them and continue doing an assigned “move” in place. The teacher moves around and checks all answers before starting over.	x			x	Flashcards
3. To the Wall	Students make lines facing the teacher, who asks a question to the first person in line. The students run to the side of the wall that represents the correct answer. Recommended to be played as a group challenge, e.g., how many students can complete the activity within one minute?”. Students waiting in line perform a different move, such as squats or jumping jacks.	x	x		x	Chalk or tape
4. Red Light	Students run in place or across the room and when the teacher calls out the “red light” word (e.g., a wrong answer) students need to stand still in a balanced position alone or with a partner.	x	x	x	x	No
5. Over/Under	Students form lines and they pass the ball to the person behind them. The last in each line runs (or skips etc.) to the board, picks a card with a question, places it in the correct pile, and runs to the front of the line passing the ball to the next person. The game continues until all cards are answered or time is up.				x	Balls or beanbags
6. Jump the Answer	Students have a personal grid with 4 options, one for each side. The teacher provides questions with multiple choice answers and students answer by jumping on the correct side of the grid. The teacher moves around and checks all answers before starting over.	x		x		Polyspots or tape
7. Messed-up Train	The teacher has created a stack of cards based on the subject (e.g., numbers that need to be placed from smaller to larger). Students line up on an assigned line on the floor and they pick one card randomly. Then they need to move to the right order on the line without losing their balance, by always placing one foot on the line.		x		x	Flashcards
8. Find your Pair	The teacher places a pile of cards (half with questions and half with answers) around the room. Students move around, with the signal they pick a card and they look for the matching card without talking. Once their pair is found they move with their partner until the teacher checks all pairs.		x		x	Flashcards

*Note*. This is a basic categorization as teachers can modify the activities by varying the setting, structure, and rules to make them developmentally appropriate for their students. Ind. = individually; Equip. = equipment.

**Table 2:** Descriptive and comparative results between M4T and Traditional lessons.

PA Levels	M4T M	M4T SD	Traditional M	Traditional SD	ES (M4T-T)
Sedentary %	40.44	19.68	42.00	27.16	−0.07
Light %	52.49	20.55	55.16	25.99	−0.12
Moderate %	5.95	8.31	2.31	2.38	0.55
Vigorous %	1.12	2.90	0.53	1.20	0.25
MVPA %	7.08	9.90	2.84	3.03	0.54
Sedentary min	18.54	9.89	18.73	12.41	−0.02
Light min	23.90	10.54	24.57	11.84	−0.06
Moderate min	2.89	4.11	1.02	1.02	0.58
Vigorous min	0.56	1.44	0.24	0.54	0.27
MVPA min	3.45	4.90	1.26	1.34	0.56

**Table 3:** Minutes of PA per MT4 activity.

M4T Activity	*N*	Sedentary min (*SD*)	Light min (*SD*)	Moderate min (*SD*)	Vigorous min (*SD*)	MVPA min (*SD*)
1. Curious Ball	5	23.20 (7.29)	19.80 (8.04)	2.60 (2.07)	4.40 (6.19)	7.00 (8.03)
2. Move Around	15	16.93 (11.37)	31.27 (11.93)	0.93 (1.94)	0.13 (0.52)	1.07 (2.28)
3. To the Wall	26	23.02 (9.48)	22.12 (9.70)	3.37 (2.95)	1.31 (3.09)	4.67 (4.51)
4. Red Light	6	24.17 (5.15)	22.00 (8.14)	2.83 (3.54)	1.00 (2.45)	3.83 (5.78)
5. Over/Under	15	21.60 (11.36)	23.53 (11.26)	3.20 (4.43)	1.67 (3.22)	4.87 (7.52)
6. Jump the Answer	16	19.69 (11.30)	29.12 (11.78)	0.75 (1.05)	0.09 (0.37)	0.84 (1.22)
7. Messed-up Train	5	25.20 (14.92)	19.80 (7.05)	4.20 (7.76)	0.80 (1.79)	5.00 (9.54)
8. Find your Pair	8	16.25 (11.23)	23.25 (11.34)	9.38 (9.96)	1.50 (2.33)	10.88 (11.87)

*Note*. 10 min of PA was recommended within a 50 min mathematics lesson. *N* represents the number of students participating in each activity.
